# Lipid raft-mediated miR-3908 inhibition of migration of breast cancer cell line MCF-7 by regulating the interactions between AdipoR1 and Flotillin-1

**DOI:** 10.1186/s12957-017-1120-9

**Published:** 2017-03-21

**Authors:** Yuan Li, Fei Shan, Jinglong Chen

**Affiliations:** 1 Department of Obstetrics and Gynecology, Beijing Obstetrics and Gynecology Hospital, Capital Medical University, Beijing, 100048 China; 2 Department of Cardiac Surgery, Affiliated Hospital of Medical College of Yan’an University, Yan’an, Shanxi 716000 China; 3 Department of Oncology, Beijing Ditan Hospital, Capital Medical University, Beijing, 100015 China

**Keywords:** MicroRNA, Breast carcinoma, Lipid raft, Migration, AdipoR1, Flotillin-1

## Abstract

**Background:**

The mechanisms of lipid raft regulation by microRNAs in breast cancer are not fully understood. This work focused on the evaluation and identification of miR-3908, which may be a potential biomarker related to the migration of breast cancer cells, and elucidates lipid-raft-regulating cell migration in breast cancer.

**Methods:**

To confirm the prediction that miR-3908 is matched with AdipoR1, we used 3’-UTR luciferase activity of AdipoR1 to assess this. Then, human breast cancer cell line MCF-7 was cultured in the absence or presence of the mimics or inhibitors of miR-3908, after which the biological functions of MCF-7 cells were analyzed. The protein expression of AdipoR1, AMPK, and SIRT-1 were examined. The interaction between AdipoR1 and Flotillin-1, or its effects on lipid rafts on regulating cell migration of MCF-7, was also investigated.

**Results:**

AdipoR1 is a direct target of miR-3908. miR-3908 suppresses the expression of AdipoR1 and its downstream pathway genes, including *AMPK*, *p-AMPK*, and *SIRT-1*. miR-3908 enhances the process of breast cancer cell clonogenicity. miR-3908 exerts its effects on the proliferation and migration of MCF-7 cells, which are mediated by lipid rafts regulating AdipoR1’s ability to bind Flotillin-1.

**Conclusions:**

miR-3908 is a crucial mediator of the migration process in breast cancer cells. Lipid rafts regulate the interactions between AdipoR1 and Flotillin-1 and then the migration process associated with miR-3908 in MCF-7 cells. Our findings suggest that targeting miR-3908 and the lipid raft, may be a promising strategy for the treatment and prevention of breast cancer.

## Background

Currently, the diagnosis of BC (breast cancer) is increasing rapidly, and has now reached epidemic proportions; half a million deaths occur from BC every year [1]. Worldwide, as a major public health problem, it negatively affects the quality of life of several millions of patients and their families [2]. Rapidly increasing incidence and prevalence rates of BC are dramatic, and are the new social challenge [3–6].

Lipid rafts orchestrate molecular interactions and are microdomains of dynamic membranes and are involved in carcinogenesis, especially BC [7]. Shah et al. have performed a general analysis on modeling progression in BC based on quantitatation of lipid rafts with proteomic datasets [7]. Their results indicated that the development of carcinoma is related to increase in the interactions between cell cytoskeleton and membrane raft; the proteins of the lipid raft constitute almost 40% of the cytoskeleton [7]. Moreover, compared with common human raft proteins, the interaction network among proteins in the simulation analysis revealed obviously higher interactions between raft proteins of cancer cells. The stabilization of domains in the lipid raft was shown to be mediated by the aforementioned evaluated cytoskeleton, and showed the reversible, functional, and common features in cancer cells through greater molecular interactions [8]. Recent researches have suggested the potential functional role associated with the raft cytoskeleton and tumor suppressors Flotillin-1 and AdipoR1.

A lipid raft is composed dynamically of lipids and proteins and numerous assembled regulatory molecules and receptors which then enable it to be regarded as the platform for signal transduction [8]. It can also form larger orderly domains, and freely float within the cellular membranes between a liquid disordered bilayer. Alterations of lipid rafts are identified and can be related to many human diseases, and microRNAs (miRNAs) can also perturb the domains of the lipid raft through targeting its proteins [9, 10]. In the past few years, the significance of the relationship between the lipid raft and BC has been elucidated [11–13].

The growing medical literature supports the vital function of miRNAs in gene expression regulation. The miRNAs are a group of tiny molecules and noncoding ribonucleic acid (RNA), 22 to 25 nucleotides in length, that function in post-transcriptional-level regulation of gene expression [14]. miRNAs control gene expression by pairing with incompletely matching target sites of the 3’-untranslated regions (UTRs) of messenger RNA (mRNA), and cause translational repression and/or mRNA destabilization, thereby downregulating the expression of the targeted gene. The regulated expression of genes is involved in numerous biological progressions, especially for the various disorders of pathogenesis (including cancer progression) [15–18]. We found that miR-3908 is expressed in the human breast cancer cell line MCF-7.

Adiponectin (APN), an adipokine produced by adipocytes, has been shown to play a critical role in the pathogenesis of obesity-associated malignancies. Through its receptor interactions, APN may exert its anti-carcinogenic effects, including regulating cell survival, apoptosis, and metastasis, via a plethora of signaling pathways [19]. Miyoshi et al. [20] first highlighted the relationship between hypoadiponectinemia and increased BC risk, where BC patients with lower APN levels are more likely to show a more aggressive phenotype. A recent meta-analysis of observational studies found that low APN levels are associated with increased risk of BC in postmenopausal, but not premenopausal women [21]. The risk reduction from higher APN levels has been reported to be around 65–80% in early BC patients [22].

To assess the role of miR-3908 in human MCF-7 cells, we first identified whether miR-3908 sequences are present in AdipoR1 mRNA. Our work firstly demonstrated that AdipoR1 is one of the targets of miR-3908 which regulates the expression of AdipoR1 followed by regulation of the AMPK/SIRT-1 signaling pathway. AdipoR1 may control the migration of MCF-7 cells mediated by the lipid raft through regulating the interaction between AdipoR1 and Flotillin-1. We, therefore, investigated the effect of miR-3908 on regulating the interaction between AdipoR1 and Flotillin-1 in MCF-7 cells. In particular, the cell migration effects of miR-3908 have been associated with the regulation of the lipid raft and the AdipoR1-AMPK/SIRT-1 signaling pathway.

## Methods

### Cell culture

MCF-7 cells were obtained from the American Type Culture Collection (ATCC), and then cultured with Dulbecco’s modified Eagle’s medium (DMEM) and fetal bovine serum (FBS) (Gibco), penicillin, and streptomycin at 37 °C in an atmosphere with 5% CO_2_.

### Extracting total RNA and cloning novel miRNA

Total cell RNA was obtained using the TRIzol method (Sigma, Palo Alto, CA, USA), based on the manufacturer’s protocol. Total RNAs were isolated with a mirVana RNA Isolation Kit and the RNA fragments smaller than 200 nt were eliminated. The miR-3908 was cloned into the open-code frame vector using a DynaExpress miRNA Cloning Kit based on its manufacturer’s instructions, and then modified.

### Reporter gene assays

The complementary deoxyribonucleic acids (cDNAs) encoding the entire 3’-UTR of AdipoR1 mRNAs were predicted and amplified from the total RNA of MCF-7 cells using *Xho* I and *Not* I linker/primers, and then cloned into the vector pmirGLO (the luciferase reporter vector) that included the gene that expresses firefly luciferase and *Renilla* luciferase. AdipoR1 3’-UTRs was cloned in reverse orientation as controls lacked the miRNA target sequence [23]. Additionally, the complementary region to the seed region of the miR-3908 sequence located in positions 139–146 of human AdipoR1 3’-UTR, CAUUGCUA. These constructs were all identified using COS-7 cells (gift from Dr. Feng Liu, Chinese Academy of Sciences), which were transfected with the reporter construct and the indicated miRNA mimic or its negative control sequences using Lipofectamine 3000 (Invitrogen, Carlsbad, CA, USA). The activity of *Renilla* luciferase was normalized with the corresponding control of the Dual-Glo® Luciferase Assay System. The mutant construct of the AdipoR1-3’-UTR was created with the Site-Mutation kit (Promega, Madison, WI, USA). Then, either negative control (NC) or miR-3908 were co-transfected into cells. Analysis of the luciferase activity of cells was detected with the VICTOR analyzer (PerkinElmer, Foster City, CA, USA). Moreover, the experiments regarding AMPK, SIRT-1, and Flotillin-1 were performed as above.

### The mimics of miR-3908 and transfection

Transfection with mimics of miR-3908 was performed using Lipofectamine 3000 (Sigma, Palo Alto, CA, USA) based on the manufacturer’s protocol. The mimics of miR-3908 were as follows: sense 5’-AAGGGAAGAUGGUGACCACUU-3’ and antisense 5’-AAGUGGUCACCAUCUUCCCUU-3’. The inhibitors of miR-3908 were as follow: 5’-GUGGUCACCAUCUUCCCUU-3’. Moreover, the NC was as follows: sense 5’-ACGUGACACGUUCGGAGAAUU-3’ and antisense 5’-AAUUCUCCGAACGUGUCACGU-3’ which was not homologous with the human genome sequences. qRT-PCR was used to identify the dose effect of miR-3908.

### qRT-PCR analysis

We identified the expression pattern of miR-3908 in MCF-7 cells according to the TaqMan miRNA assays with its specific primers. The 2^−ΔΔCt^ method was used to analyze the data. qRT-PCR was performed with a SYBR Green kit (Sigma, Palo Alto, CA, USA). The primer sequences were as follows: AdipoR1 upstream 5’-**CAGATTTTCCATGTCCTGGTG**-3’ and downstream 5’-**CGGAATTCCTGAAGGTTGG**-3’; and glyceraldehyde-3-phosphate dehydrogenase (GAPDH) upstream 5’-CTCATGACCACAGTCCATGCC-3’and downstream 5’-GGCATGGACTGTGGTCATGAG-3’.

### Western blotting

Cells were lysed, and then total proteins were extracted with radioimmunoprecipitation assay (RIPA) lysis buffer. Total proteins were analyzed with an electrophoresis method using sodium dodecyl sulfate-polyacrylamide (SDS-PAGE) gel, and then transferred to a polyvinylidene fluoride (PVDF) membrane with a 0.45-μm pore size (Roche, Branchburg, NJ, USA). This process was prohibited with 5% skimmed milk and the preparation then washed three times with Tris-buffered saline (TBST) at room temperature, and then at 4 °C, and probed with the antibodies: AdipoR1, SIRT-1, AMPK (1:4000, Cell Signaling, Danvers, MA, USA), p-AMPK, Flotillin-1, β-actin (1:2000, Santa Cruz Biotechnology, Santa Cruz, CA, USA) overnight. Finally, the preparation was incubated at room temperature with proper secondary antibodies (1:5000 dilutions, Santa Cruz Biotechnology, Santa Cruz, CA, USA).

### Analysis of proliferation and clonogenicity in MCF-7 cells

Cell proliferation ability was detected with an MTS assay. With crystal violet, the colonies of cells were stained, and then fixed by formalin. The clonogenicity assay could demonstrate the quantity of developed cell colonies which differs from other methods.

### Cell migration assay

The migration ability of MCF-7 cells was measured using a wound scratch assay. Cells were first scratched, and then their movements were observed and measured at 48 h. The migratory cells were counted and their total number quantified.

### Co-immunoprecipitation

Five microliters phenylmethanesulfonyl fluoride (PMSF) was added to the cultured cells, and then the cells were lysed. To each supernatant from the cell extraction was added 50 μL Protein G Plus/Protein A Agarose Suspension (Calbiochem, San Diego, CA, USA). Ten microliters anti-AdipoR1 or anti-Flotillin-1 antibody was added into the supernatants which were rotated gently overnight, respectively. Sixty microliters Protein G Plus/Protein A Agarose Suspension was then added to the samples, which were then rotated gently for 9 h at 4 °C and finally centrifuged for 5 min. Immunoprecipitates were washed and resuspended with loading buffer before boiling or electrophoresis, then resolved by SDS-PAGE; anti-Flotillin-1 and anti-AdipoR1 antibody were used for immunoblotting, respectively.

### Confocal laser scanning

The cDNA sequences of AdipoR1 and Flotillin-1 were cloned into the pDS-RED1-N1 or pEGFP-C1 expression vector, respectively. The pEGFP-C1-Flotillin-1 and pDS-RED1-N1-AdipoR1 vectors were then co-transfected into MCF-7 cells. After transfection for 48 h, cells were treated with 4% paraformaldehyde, and then stained with DAPI (4’,6-diamidino-2-phenylindole). These cells were then observed with a LSM 510 microscope (Zeiss, Oberkochen, Germany).

### Purification of membrane and lipid raft protein

Membrane proteins were isolated from MCF-7 cells by a multiple-centrifugation procedure [24]. Lipid raft fractions were purified from MCF-7 cells by a modified sucrose density gradient ultracentrifugation procedure [25, 26]. Briefly, MCF-7 cells were lysed with ice-cold MBS buffer. The lysates were mixed with sucrose and ultracentrifuged for 18 h at 39,000 rpm, then the samples were separated to 12 subfractions [27].

### Statistical analyses

Statistical differences between groups were analyzed with a two-tailed, paired Student *t* test. Data for qRT-PCR and luciferase reporter assays were expressed relative to the control in each experiment, and 95% confidence intervals were calculated. Continuous variables were shown with mean ± SD (standard deviation). Between groups, abnormally distributed data were analyzed using a Kruskal-Wallis analysis of variance (ANOVA). All statistical analyses were achieved using SPSS software (SPSS Inc., Chicago, IL, USA; version 18.0). *P* < 0.05 was regarded as statistically significant.

## Results

### Expression of AdipoR1 was suppressed by miR-3908 in MCF-7 cells

**Fig. 1:**
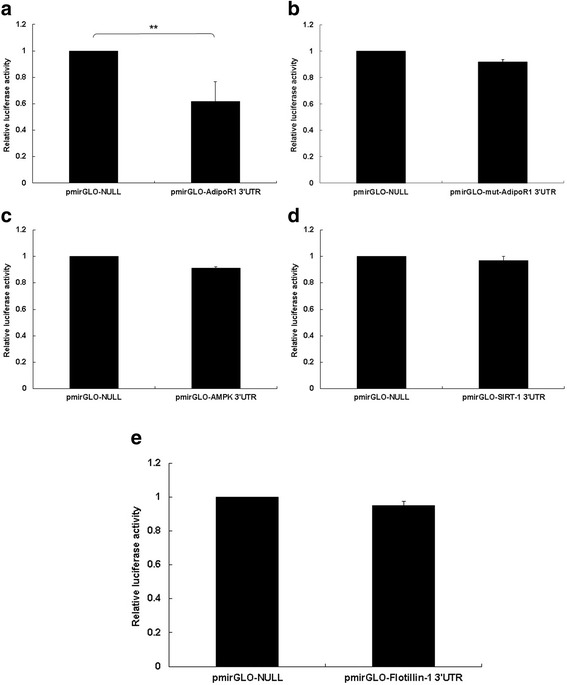
Expression of AdipoR1 was suppressed by miR-3908 in MCF-7 cells. To study if microRNA modulates AdipoR1, or the downstream pathway of AdipoR1 in MCF-7 cells, firstly, using the online software Targetscan (http://targetscan.org/), we tried to forecast miRNAs that target AdipoR1. Within these miRNAs, miR-3908 potentially co-targeted mRNAs of AdipoR1 3’-UTRs. A further proof experiment was carried out with a luciferase reporter gene assay to identify whether 3’-UTRs of AdipoR1 were bonded directly with miR-3908. The results showed that the relative luciferase activities of AdipoR1 3’-UTR were obviously downregulated in MCF-7 cells transfected with miR-3908 (**a**), but there was no change in the mut-AdipoR1 group (**b**). However, it was identified that miR-3908 could not form direct bonds with AMPK (**c**), SIRT-1 (**d**), or Flotillin-1 (**e**) 3’-UTRs, respectively. The data are presented as means ± SD from three independent experiments. *P < 0.05, **P < 0.01

To explore whether miRNA regulates AdipoR1, or the upward pathway of AdipoR1 in MCF-7 cells, firstly we used the online software Targetscan (http://targetscan.org/) to forecast miRNAs that target AdipoR1. Within these miRNAs, miR-3908 potentially co-targeted the mRNAs of AdipoR1 3’-UTRs. A further proof experiment was carried out with a luciferase reporter gene assay, to identify whether 3’-UTRs of AdipoR1 were bonded directly with miR-3908. The results showed that the relative luciferase activities of AdipoR1 3’-UTR were obviously downregulated in MCF-7 cells transfected with miR-3908 (Fig. 1a), but there was no change in the mut-AdipoR1 group (Fig. 1b). However, it was identified that miR-3908 could not form direct bonds with AMPK (Fig. 1c), SIRT-1 (Fig. 1d), or Flotillin-1 (Fig. 1e) 3’-UTRs, respectively. Our work identified that miR-3908 could directly target the mRNAs of AdipoR1.

**Fig. 2:**
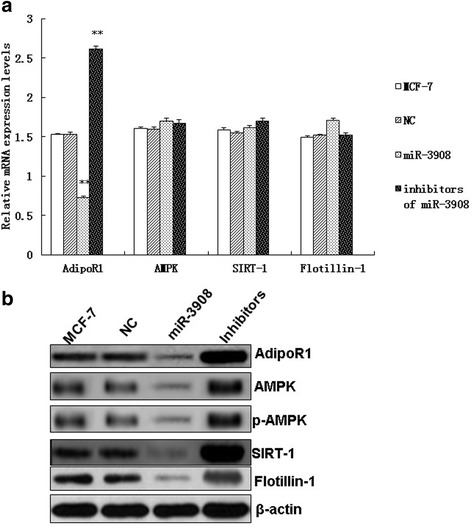
Expression of AdipoR1 was suppressed by miR-3908 in MCF-7 cells. To verify the miR-3908 effect on the mRNA and protein expression of AdipoR1, AMPK, SIRT-1, and Flotillin-1 in MCF-7 cells, we accomplished relative quantification analyses with qRT-PCR and Western blots, respectively. miR-3908 inhibited the mRNA expression of AdipoR1 in MCF-7 cells (**a**), as well as the protein expression of AdipoR1 (**b**). Moreover, miR-3908 did not affect the mRNA expression of AMPK, SIRT-1, and Flotillin-1, but the protein expression of AMPK, p-AMPK, SIRT-1, and Flotillin-1 also decreased (**b**). Furthermore, the inhibitors of miR-3908 promoted the mRNA expression of AdipoR1 in MCF-7 cells, but did not affect the mRNA expression of AMPK, SIRT-1, and Flotillin-1 (**a**). Altogether, our results demonstrated that miR-3908 directly regulates the expression of AdipoR1 by interacting with its 3’-UTR, and then the protein expression of its downstream genes. The data are presented as means ± SD from three independent experiments. *P < 0.05, **P < 0.01

To confirm the miR-3908 effect on the mRNA and protein expression of AdipoR1, AMPK, SIRT-1, and Flotillin-1 in MCF-7 cells, we accomplished relative quantification analyses with qRT-PCR and Western blots, respectively (Fig. 2). The results indicated that miR-3908 inhibited the mRNA expression of AdipoR1 in MCF-7 cells (Fig. 2a), as well as the protein expression of AdipoR1 (Fig. 2b). Moreover, miR-3908 did not affect the mRNA expression of AMPK, SIRT-1, and Flotillin-1, but the protein expression of AMPK, p-AMPK, SIRT-1, and Flotillin-1 also decreased (Fig. 2b). Furthermore, the inhibitors of miR-3908 promoted the mRNA expression of AdipoR1 in MCF-7 cells, but did not affect the mRNA expression level of SIRT-1, AMPK, and Flotillin-1 (Fig. 2a). Altogether, our results demonstrated that miR-3908 directly regulates the expression of AdipoR1 by interacting with its 3’-UTR, and then the protein expression of its downstream genes.

### miR-3908 suppresses cell growth and clonogenicity in MCF-7 cells

**Fig. 3:**
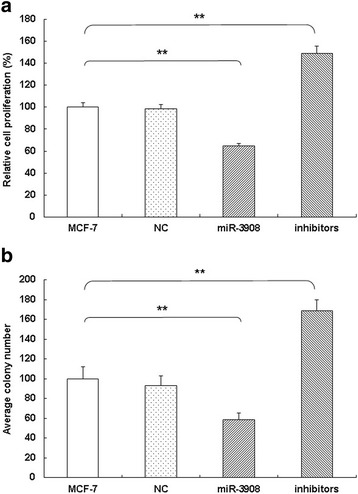
miR-3908 suppresses cell growth and clonogenicity in MCF-7 cells. For evaluating the overexpressed or underexpressed possible biotic outcomes induced by miR-3908, several functional experiments were performed in MCF-7 cells. Compared with control cells, proliferation of MCF-7 cells (**a**) would be suppressed by transfection with miR-3908. Moreover, the inhibitors of miR-3908 accentuated the same process in MCF-7 cells (**a**). Furthermore, the colony formation assay revealed that miR-3908 played a significant role in the suppression of this ability compared with the control group in MCF-7 cells. However, downregulation of miR-3908 facilitated significantly the colony-forming ability of MCF-7 cells. These results suggested that miR-3908 also lowered colony formation of MCF-7 cells (**b**) (*P* < 0.001). The data are presented as means ± SD from three independent experiments. *P < 0.05, **P < 0.01

For evaluating the overexpressed or underexpressed possible biotic outcomes induced by miR-3908, several functional experiments were performed in MCF-7 cells. Compared with control cells, proliferation of MCF-7 cells (Fig. 3a) would be inhibited when they were transfected with miR-3908. Moreover, the inhibitors of miR-3908 accentuated the same process of MCF-7 cells (Fig. 3a). These results demonstrated that miR-3908 suppressed the proliferation of MCF-7 cells.

Furthermore, the colony formation assay revealed that miR-3908 played a significant role in the suppression of proliferative ability compared with the control group in MCF-7 cells. However, downregulation of miR-3908 facilitated significantly the colony-forming ability of MCF-7 cells. These results suggested that miR-3908 also lowered colony formation of MCF-7 cells (Fig. 3b) (*P* < 0.001).

### miR-3908 suppresses migration of MCF-7 cells

**Fig. 4:**
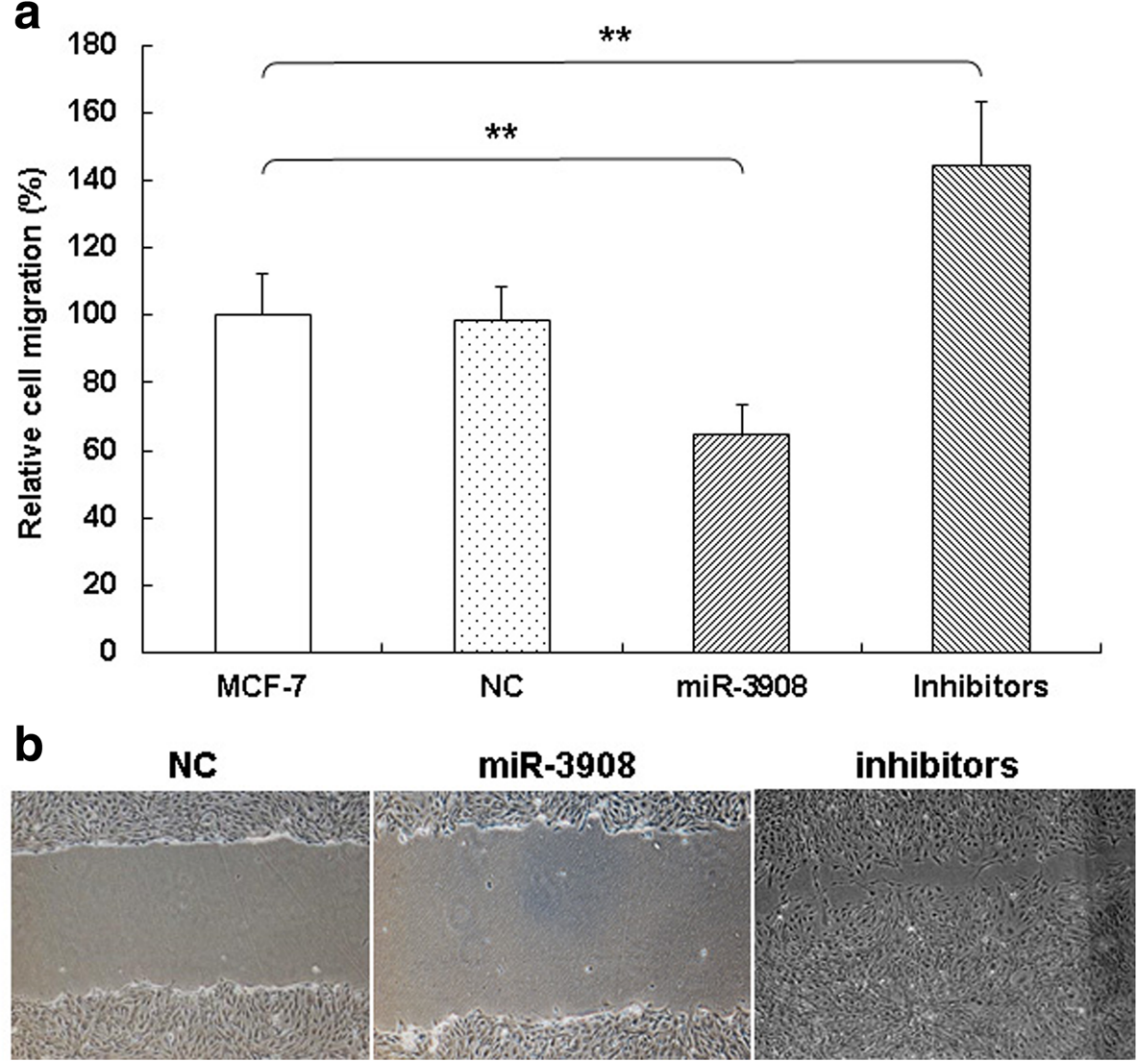
miR-3908 suppresses migration of MCF-7 cells. miR-3908 inhibited significantly the migration ability of MCF-7 cells compared to control (NC) (**a** and **b**) in vitro (*P* < 0.001). Conversely, using the inhibitors of miR-3908, the migration ability was enhanced in MCF-7 cells (**a** and **b**). These results suggested that miR-3908 suppresses the migration of MCF-7 cells. The data are presented as means ± SD from three independent experiments. *P < 0.05, **P < 0.01

miR-3908 inhibited significantly the migration ability of MCF-7 cells compared to control in vitro (Fig. 4; *P* < 0.001). Conversely, using the inhibitors of miR-3908, the migration ability was enhanced in MCF-7 cells (Fig. 4). These results suggested that miR-3908 suppresses the migration ability and invasion of MCF-7 cells.

### AdipoR1 protein interacted with Flotillin-1

**Fig. 5:**
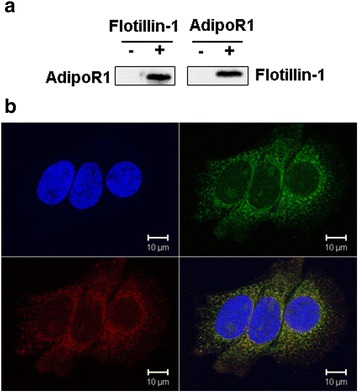
AdipoR1 protein interacted with Flotillin-1. The proteins that interacted with AdipoR1 were analyzed by co-immunoprecipitation (CO-IP) and confocal laser scanning microscopy (CLSM). MCF-7 cells were harvested after 48 h in culture, then total cellular proteins were extracted and CO-IP’d (co-immunoprecipitated) with anti-AdipoR1 antibodies followed by anti-Flotillin-1 antibodies (**a**). Then, the above proteins were then Co-IP’d with anti-Flotillin-1 antibodies followed by AdipoR1 antibodies (**a**). AdipoR1 is localized to the cell membrane of EC. After MCF-7 cells were co-transfection with plasmids pEGFP-C1-Flotillin-1 (*green*) and pDS-RED1-N1-AdipoR1 (*red*) for 48 h, the confocal scanning images demonstrated that the Flotillin-1 recombinant protein was localization to the cell membrane. The nuclei of the cells (*blue*) were stained by DAPI. The overlaid image indicates that AdipoR1 overlapped with Flotillin-1 at the MCF-7 cell membrane (**b**)

**Fig. 6:**
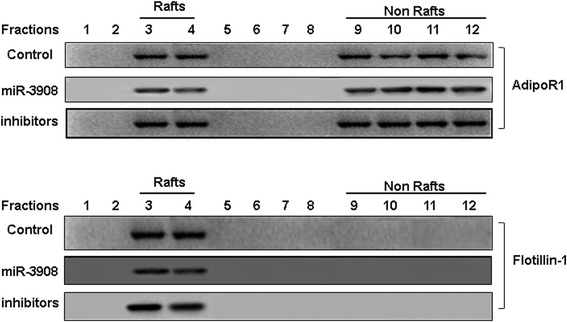
The inhibitors of miR-3908 induced the mobility of AdipoR1 into lipid rafts. In the last few years, many researches have indicated that functional receptors assemble at the platforms provided by lipid rafts. In metastasis and progression of breast cancer (BC), AdipoR1 plays an important role, and may be a potential alternative therapeutic goal for patients with BC. The migration of AdipoR1 into lipid rafts plays an important role in the anti-invasion process. To explore the anti-invasion functions of miR-3908 inhibitors, the role of these inhibitors on the mobility of AdipoR1 into lipid rafts was determined. Our results demonstrated that miR-3908 inhibitors reduced the migration of AdipoR1 into fractions of the lipid raft. The phenomenon could be enhanced by miR-3908, but reversed by inhibitors of miR-3908 as well as by Flotillin-1

The proteins that interacted with AdipoR1 were analyzed by CO-IP (co-immunoprecipitation) and confocal laser scanning microscopy (CLSM). MCF-7 cells were harvested after 48 h in culture, then total cellular proteins extracted and CO-IP’d (co-immunoprecipitated) with anti-AdipoR1 antibodies followed by anti-Flotillin-1 antibodies (Fig. 5a). Then, the above proteins were then Co-IP’d with anti-Flotillin-1 antibodies followed by AdipoR1 antibodies (Fig. 6a). AdipoR1 is localized to the cell membrane of MCF-7 cells. After MCF-7 cells were co-transfected with plasmids pEGFP-C1-Flotillin-1 (green) and pDS-RED1-N1-AdipoR1 (red) for 48 h, the confocal scanning images demonstrated that the Flotillin-1 recombinant protein was localization to the cell membrane. The nuclei of the cells (blue) were stained by DAPI. The overlaid image indicates that AdipoR1 overlapped with Flotillin-1 at the cell membrane (Fig. 5b). These results demonstrated that AdipoR1 protein may interact with Flotillin-1 at the cell membrane of MCF-7 cells.

### The inhibitors of miR-3908 induced mobility of AdipoR1 into lipid rafts

In the last few years, many researches have indicated that functional receptors assemble at the platforms provided by lipid rafts. In metastasis and progression of BC, AdipoR1 plays an important role, and may be a potential alternative therapeutic goal for patients with BC [28]. The migration of AdipoR1 into lipid rafts plays an important role in anti-invasion process. To explore the anti-invasion functions of miR-3908 inhibitors, the role of these inhibitors on the mobility of AdipoR1 into lipid rafts was determined. Our results demonstrated that miR-3908 inhibitors reduced the migration of AdipoR1 into fractions of the lipid raft. The phenomenon could be enhanced by miR-3908, but reversed by inhibitors of miR-3908 as well as by Flotillin-1 (Fig. 6).

## Discussion

The mechanism for the transcriptional control of gene expression associated with BC has been explored. Although it is well known that there are several different angiogenetic mechanisms, it is still unclear how miRNA modulates lipid raft-mediated angiogenesis. A number of factors, such as miRNAs, play a crucial role in modulating gene expression, such as those involved in BC [29].

miRNAs are involved in the physiological processes of cells; however, deregulation of them may be related to cancer. Many miRNAs in BC exhibit a different expression level from normal human breast tissues. Furthermore, in BC, a few miRNAs have been identified that are correlated with tumor subtype, therapy response, and pathology phenotype. As drug resistance in patients with BC increases, miRNAs are becoming the potential biomarker for the prediction of fresh targets for BC treatment. In the present work, we ascertained the novel miRNA, miR-3908, and investigated its effects and meanings in the developmental process of BC.

Adipose tissue secretes an adipokine called adiponectin, which is downexpressed in obesity with insulin-resistance. Its biological effects are performed by its receptors AdipoR1 and AdipoR2. AdipoR1 may upregulate the expression of AMPK (adenosine monophosphate-activated protein kinase), and PPAR (peroxisome proliferator-activated receptor)-α may be activated by AdipoR2 [30]. However, the miRNA-mediated regulation of AdipoR1 activity during the developmental process of BC remains poorly understood.

As shown in the current study, our bioinformatics analysis, using online software (http://targetscan.org/), confirmed that the 3’-UTR site of AdipoR1 mRNA is complimentary to miR-3908. The results of luciferase reporter analysis indicated that the relative activity of AdipoR1 3’-UTR significantly decreases in MCF-7 cells transfected with miR-3908, but there were no changes in the mut-AdipoR1 group. Moreover, it was identified that miR-3908 could not form direct bonds with AMPK, SIRT-1, or Flotillin-1 3’-UTRs, respectively. Following overexpression of miR-3908 in MCF-7 cells compared to NC, Western blot analyses verified that protein expression levels of AdipoR1 downregulated in MCF-7 cells induced by miR-3908. Furthermore, miR-3908 did not affect the mRNA expression of AMPK, SIRT-1, and Flotillin-1, but the protein expression of AMPK, p-AMPK, SIRT-1, and Flotillin-1 also decreased. These outcomes verified that the mRNA of AdipoR1 is the direct target of miR-3908, and suppresses protein expression of its downstream pathway genes, including AMPK, p-AMPK, and SIRT-1. Therefore, this provides a new therapy target for BC treatment.

For evaluating the possible biotic outcomes induced by miR-3908, we overexpressed or underexpressed miR-3908, and then performed a few functional experiments related to the molecular functions of MCF-7 cells. Subsequently, MCF-7 cells were exposed to the mimics or inhibitors of miR-3908, and then the cell functions of MCF-7 cells, such as proliferation, colony formation, and migration, were analyzed. These results demonstrated that miR-3908 suppressed the proliferation of MCF-7 cells, lowered its colony formation, and restrained its ability to influence cell migration.

The changes in BC cells conducive to tumor evolution, heterogeneity, metastasis, and ultimately, drug resistance are shaped by numerous genetic changes including alterations in cellular metabolism. These include intermediary metabolic pathways such as amino acid synthesis, oxidative phosphorylation, the citric acid cycle, glycolysis, and lipid metabolism [31].

There is extensive evidence showing that the metabolism of both exogenous and endogenous substances influences BC growth. Moreover, it is clear that metabolism can influence each unique BC subclass in diverse ways and to varying degrees. These metabolic changes have been categorized in an assortment of ways; for example, many involve changes in energy metabolism such as in the way that glucose, amino acids, and lipids are processed [31]. Profound advances have been made towards comprehending the changes in genetic information that occur during BC development, particularly in the area of cancer metabolism. Furthermore, cancer metabolism traditionally refers to intermediary metabolism and generally does not include other cellular metabolic-related pathways and processes such as drug metabolism. However, alterations in drug-metabolizing enzymes and metabolite transporters play important roles in oncogenesis and drug resistance. New insights into the differential expression patterns of metabolic enzymes between ER-positive and ER-negative BC have the strong potential to guide rational development of novel therapeutic agents and to reshape current treatment regimens for optimal outcomes [31].

In addition, the expression patterns of many metabolic enzymes change as tumors become more aggressive and metastatic. Although, it is less clear, it appears that chemotherapy alters the metabolic phenotype of cancer cells which may offer a therapeutic opportunity [32]. Therefore, novel agents and approaches are needed that harness metabolic differences in BC which set them apart from normal tissues. For example, delivering small molecules or peptides via nanoparticles is an exciting therapeutic approach because of their ability to target specific cell types and organelles such as mitochondria [33–35]. Paulmurugan et al. recently published a report where they delivered the FASN (fatty acid synthase) inhibitor orlistat to TNBC (triple-negative breast cancer) via folate-decorated polymeric nanoparticles leading to enhanced cytotoxicity [36]. Another emerging therapeutic target in BC that may account for the intratumoral heterogeneity seen in patients’ tumors are BCSCs (breast cancer stem cells). As noted, BCSCs are heavily glycolytic and may influence the metabolic phenotype of the tumor. A variety of pharmacologic agents for targeting these cells are at varying stages of development.

MiRNAs play a significant role in the functional regulation of MCF-7 cells. MiRNAs are coincidentally mentioned to modulate genetic expression in all types of cancer cells by regulating extensive processes in biology related to the progression and development of tumors such as cell differentiation, apoptosis, and proliferation. Previous studies indicate that miRNAs act as oncogenic or oncosuppressive miRNA to promote or inhibit BC tumorigenesis and progression, and may be conducive to the diagnosis and treatment of BC [37]. Lipid rafts make up membrane microdomains which are the platforms of a kind of signaling molecule. Compared to the surrounding membrane, these microdomains are more tightly packed and ordered [38]. In recent times, many researches have demonstrated that many functional receptors were assembled on the platforms provided by lipid rafts [39]. Flotillin-1 protein localizes to caveolae and plays an important role in cell morphology and vesicle trafficking [40]. The proteins that interacted with AdipoR1 were analyzed by CO-IP and CLSM. Our results indicated that AdipoR1 protein may interact with Flotillin-1 at the cell membrane of MCF-7 cells.

It is known that AdipoR1 plays an important role in, and may be a potential alternative therapeutic goal for, patients with BC. The migration of AdipoR1 into lipid rafts play an important role in the anti-migration process. Our results demonstrated that miR-3908 inhibitors reduced the mobility of AdipoR1 into the fractions of the lipid raft. Moreover, miR-3908 could enhance its migration, but the phenomenon was reversed by inhibitors of miR-3908 as well as by Flotillin-1. miR-3908 suppressed the interaction between AdipoR1 and Flotillin-1 by inhibiting the expression of the AdipoR1 or AMPK/SIRT1 signaling pathway. Furthermore, the packages of lipid rafts were blocked and migration of MCF-7 cells was restrained.

## Conclusions

In summary, we have illustrated miR-3908, which specifically binds to AdipoR1 mRNA 3’-UTR. miR-3908 has effects on the biological functions of MCF-7 cells such as cell proliferation, colony formation, and migration. Moreover, AdipoR1 is a crucial mediator of migration in MCF-7 cells through inhibiting the AMPK/SIRT-1 signaling pathway. Lipid rafts regulate the interactions between AdipoR1 and Flotillin-1, and then the migration process associated with miR-3908 in MCF-7 cells. Our findings suggest that targeting miR-3908 and the lipid raft, being involved in BC cell migration, is a hopeful method for the prevention and treatment of BC.

## Abbreviations

3’-UTRs: 3’-untranslated regions
AMPK: Adenosine monophosphate-activated protein kinase
APN: Adiponectin
BC: Breast cancer
BCSCs: Breast cancer stem cells
CLSM: Confocal laser scanning microscopy
CO-IP’d: Co-immunoprecipitated
FASN: Fatty acid synthase
miRNAs: MicroRNAs
NC: Negative control
PMSF: Phenylmethanesulfonyl fluoride
PPAR: Peroxisome proliferator-activated receptor
PVDF: Polyvinylidene fluoride
TNBC: Triple-negative breast cancer.
